# MiR-144-3p regulates cell proliferation and apoptosis in renal ischemia-reperfusion injury by targeting EZH2

**DOI:** 10.3389/fmed.2026.1745179

**Published:** 2026-07-02

**Authors:** Lifeng Zhang, Fuxin Zheng, Xin Li, Rui Min, Yaping Yang, Wei Jiang, Lei Lyu

**Affiliations:** 1 Department of Urology, Ezhou Centre Hospital, Ezhou, China; 2 Department of Urology, Wuhan No.1 Hospital (Traditional Chinese and Western Medicine Hospital of Wuhan), Tongji Medical College, Huazhong University of Science and Technology, Wuhan, China

**Keywords:** apoptosis, Bax, Caspase-3, EZH2, ischemia-reperfusion injury, miR-144-3p

## Abstract

Renal ischemia-reperfusion injury (IRI) is a leading cause of acute kidney injury, with limited effective therapeutic options. While microRNAs (miRNAs) play crucial roles in regulating various diseases, including renal IRI, their specific targets and mechanisms remain elusive. In this study, hypoxia/reoxygenation (H/R) significantly promoted apoptosis in both NRK-52E and HK-2 cells, accompanied by a marked downregulation of miR-144-3p in both H/R-treated cells and the kidneys of mice subjected to ischemia/reperfusion (I/R) injury. Moreover, miR-144-3p overexpression enhanced cell viability under H/R conditions, as evidenced by the results of MTT assays. Dual-luciferase reporter assays identified *EZH2* as a direct target of miR-144-3p, while Western blot analysis revealed that miR-144-3p suppressed EZH2 protein expression. Furthermore, *EZH2* knockdown reduced *Bax* and cleaved Caspase-3 expression, highlighting its role in apoptosis regulation. Finally, miR-144-3p protected against renal IRI by inhibiting apoptosis and promoting cell proliferation by modulating EZH2, offering novel insights into its protective mechanisms. These findings position EZH2 as a potential therapeutic target for mitigating renal IRI.

## 1 Introduction

As is well documented, renal ischemia-reperfusion injury (IRI) is one of the leading causes of acute kidney injury, for which effective therapeutic strategies remain limited (1). Several factors, including oxidative stress (2), energy metabolism (3), cell apoptosis (4) and inflammation (5), have been implicated in the pathogenesis of renal IRI (6, 7). The kidneys possess unique physiological structures and functions that render them susceptible to ischemia and hypoxia (8). As a result, patients with renal (IRI) face a significantly higher risk of acute kidney injury (AKI) and mortality (9). During acute kidney injury, renal blood flow temporarily decreases and is followed by a reperfusion phase (10). Although reperfusion is critical for the restoration of kidney function, it is also associated with substantial cellular injury (11). However, renal IRI is an unavoidable complication during kidney transplantation and nephron-sparing partial nephrectomy, and its pathogenesis is highly intricate (12). Therefore, there is a pressing need to investigate the molecular mechanism underlying renal IRI to develop effective strategies for the prevention and treatment of renal IRI and AKI.

MicroRNAs (miRNAs) are small non-coding RNAs approximately 22 nucleotides in length that regulate gene and protein expression by post-transcriptionally binding to the 3′ untranslated region (UTR) of target mRNAs, thereby repressing translation and/or inhibiting protein synthesis (13). They have been extensively investigated as powerful regulatory factors in various diseases, potentially playing a decisive role in the onset and/or progression of conditions such as kidney disease. For instance, miR-132-3p has been reported to aggravate renal ischemia-reperfusion injury by targeting the Sirt1/PGC1alpha axis (14), whilst miR-10a exacerbated renal ischemia-reperfusion injury associated with decreased PIK3CA expression (15). Conversely, miR-29a-3p has been described to alleviate renal ischemic injury by targeting TNFR1 and collagen I (16). At the same time, miR-24 has been reported to promote renal ischemic injury by stimulating apoptosis in endothelial and tubular epithelial cells (17). Besides, Chenguang Ding et al. concluded that miR-124 functioned as a negative regulator of endoplasmic reticulum stress (ERS) by interacting with IRE-1α, ultimately exerting renoprotective effects (18). These reports highlight the clinical relevance of these miRNAs in renal IRI. Although certain miRNAs have been identified to exert protective effects in renal IRI, the underlying mechanisms remain to be elucidated. Mounting data suggests that exosome-mediated miR-144-3p promoted ferroptosis and concurrently inhibited the proliferative, migratory, and invasive abilities of osteosarcoma cells by regulating ZEB1 (19). miR-144-3p was sponged by LncRNA TUG1 to attenuate ischemia-reperfusion-induced apoptosis of renal tubular epithelial cells via targeting Nrf2 (20). However, the mechanisms by which miR-144-3p influences renal IRI remain underexplored, warranting additional research to delineate its functions.

The enhancer of zeste homolog 2 (EZH2) serves as a significant substrate for Smurf2, playing essential roles in various cellular processes, including cell division, autophagy, embryonic development, and signal transduction (21). In an earlier study, targeting EZH2 with BMSCs-exo loaded with miR-367-3p protected mouse skeletal muscles against pyroptosis-induced ischemia/reperfusion (I/R) injury (22). Additionally, EZH2 was directly bound by lncRNA JPX, leading to a reduction in EZH2-mediated H3K27me3 modifications in the SERCA2a promoter region, thereby protecting the heart from acute myocardial ischemia/reperfusion (I/R) injury (23). This activation protects cells against IRI. However, the involvement of EZH2 in renal IRI, including its specific roles and underlying mechanisms, remains poorly understood.

Thus, the present study aimed to investigate the role of miR-144-3p in renal I/R injury. The results revealed that miR-144-3p expression was downregulated following renal I/R. Moreover, miR-144-3p overexpression inhibited cell apoptosis and promoted cell proliferation, thereby alleviating renal I/R injury. Mechanistically, miR-144-3p directly bound to *EZH2* 3’ UTR and suppressed its translation, which led to the downregulation of *Bax* and cleaved Caspase-3. Besides, this study uncovered novel molecular mechanisms underlying renal I/R injury, which could assist in the identification of therapeutic targets. Furthermore, this study examined the role of miR-144-3p in renal ischemia-reperfusion (I/R) injury.

## 2 Materials and methods

### 2.1 Cell culture, model, and transfection

The rat renal epithelial cell line NRK-52E(RTEC), rat renal fibroblast NRK-49F, and human renal proximal tubular epithelial cell line HK-2, all purchased from American Type Culture Collection (ATCC), were cultured in Dulbecco’s Modified Eagle Medium/F12 (DMEM/F12; Gibco, Carlsbad, CA) supplemented with 10% fetal bovine serum, 10 U/mL penicillin, and 10 U/mL streptomycin (Gibco, Carlsbad, CA) at 37°C in a humidified incubator (Thermo Fisher Scientific, Marietta, OH). To establish an NRK-52E cell model of renal IRI via hypoxia/reoxygenation, the cells were cultured in serum-free medium under hypoxic conditions (94% N_2_, 5% CO_2_, and 1% O_2_) in a tri-gas incubator for 24 h, followed by reoxygenation (95% air and 5% CO_2_) for 4 h (18). For reoxygenation, the hypoxic medium was replaced, and cells were returned to normoxic conditions (5% CO_2_ at 37°C) for 0_−_12 h, depending on experimental requirements.

Prior to transfection, cells were seeded in six-well plates and cultured for 24 h. Transfection was performed using Lipofectamine™ 2000 (Invitrogen, Carlsbad, CA, United States) following the manufacturer’s protocol. *EZH2* shRNA (Si-EZH2) was synthesized by Guangzhou RiboBio Co., Ltd, and miR-144-3p mimics and inhibitors were purchased from Genepharma (Shanghai, China). The sequences of miRNA mimics, inhibitors, and primers are listed in Supplementary Table 1.

### 2.2 Animals and IRI model

All animal experiments were approved by the Animal Care Committee of Wuhan No.1 Hospital, Tongji Medical College, Huazhong University of Science and Technology. Six C57BL/6 wild-type mice were assigned to each group and housed at the Animal Experimental Center, Huazhong University of Science and Technology. To establish a mouse model of renal IRI *in vivo*, male mice aged 8–10 weeks and weighing 20–25 g were intraperitoneally anesthetized with sodium phenobarbital (60 mg/kg). Next, bilateral renal pedicles were clamped to induce ischemia for 30 min, followed by 0–48 h of reperfusion, depending on experimental requirements. Mice in the sham group underwent identical surgical procedures without clamping.

### 2.3 Histopathological assessment

Briefly, freshly excised murine kidney tissues were fixed in 4% paraformaldehyde at room temperature for 16 h. Following fixation, the tissues were dehydrated through a graded ethanol series and embedded in paraffin. Afterward, the paraffin-embedded sections were sectioned at a thickness of 5 μm using a microtome. Following this, they were deparaffinized in xylene, rehydrated through a descending ethanol gradient, and rinsed with distilled water. Hematoxylin staining was carried out for 5 min, followed by rinsing with water, differentiation for 30 s, and counterstaining with eosin for 30 s at room temperature. Histological evaluation was performed in a blinded manner under light microscopy. Tubular injury was semi-quantitatively scored based on the percentage of affected tubules exhibiting epithelial necrosis, luminal necrotic debris, tubular dilation, or hemorrhage. The scoring criteria were as follows: 0, no detectable damage; 1, mild (< 25% involvement); 2, moderate (25–50%); 3, severe (51–75%); and 4, extensive (> 75%), as described in a previous study (24).

### 2.4 Detection of oxidative stress-related markers

The extent of lipid peroxidation and antioxidant enzyme activity was assessed by quantifying malondialdehyde (MDA) levels and superoxide dismutase (SOD) activity, respectively. Both assays were performed using specific commercial kits (MDA, G4302-96T; SOD, G4306-96T; Servicebio, Wuhan, China) according to the manufacturer’s protocols. For MDA detection, the thiobarbituric acid reactive substances (TBARS) method was employed. Tissue homogenates were incubated with thiobarbituric acid under acidic conditions, and the resulting pink chromogen was measured spectrophotometrically at 532 nm. MDA concentration was calculated using a standard curve and expressed as nmol per mg protein. SOD activity was determined based on its ability to inhibit the superoxide-dependent reduction of a water-soluble tetrazolium salt. The reduction product was quantified colorimetrically at 450 nm. One unit of SOD activity was defined as the amount of enzyme required to cause 50% inhibition of the reduction reaction under the assay conditions, and results were normalized to protein content. All samples were analyzed in triplicate, and the complete experiment was independently repeated three times to ensure reproducibility.

### 2.5 Flow cytometry

After 24 h of culture, cells were collected and washed with pre-cooled 1 × PBS at 4°C. They were then resuspended in 200 μL of 1 × binding buffer, followed by the introduction of 5 μL of FITC for labeling at 37°C for 15 min. Subsequently, 150 μL of 1 × binding buffer and 5 μL of propidium iodide (PI) were added for cell staining. All stained cells were subsequently analyzed using flow cytometry (FACS Calibur; BD Biosciences, Franklin Lakes, NJ, United States).

### 2.6 Dual-luciferase reporter assay

Dual-luciferase reporter assays were performed to evaluate both the post-transcriptional regulation of *EZH2* by miR-144-3p and the transcriptional activity of *EZH2* on the pro-apoptotic genes *Bax* and cleaved Caspase-3. In the miRNA-target interaction assay, wild-type and mutant sequences of the *EZH2* 3′ untranslated region (3′UTR) containing the predicted miR-144-3p binding sites were cloned into the pmirGLO Dual-Luciferase miRNA Target Expression Vector (Promega, Madison, WI, United States). NRK-52E cells were co-transfected with these constructs and miR-144-3p mimics or negative control mimics using Lipofectamine 2000 (Invitrogen, Carlsbad, CA, United States). In the promoter activity assay, approximately 1,000 bp upstream promoter regions of *Bax* and cleaved Caspase-3 were amplified and inserted into the pGL3-basic luciferase vector (Promega, Madison, WI, United States). Cells were co-transfected with the promoter constructs and either *EZH2* siRNA or control siRNA. In both assays, luciferase activity was measured 48 h post-transfection using the Dual-Luciferase^®^ Reporter Assay System (Omega, United States) on an Omega plate reader. Firefly luciferase activity was normalized to Renilla luciferase activity.

### 2.7 Western blot analysis

Total protein was extracted, and concentrations were detected as previously described. Antibodies against EZH2 (1:8,000, Cat. No: 21800-1-AP), BAX (1: 30,000, Cat. No: 50599-2-Ig), cleaved Caspase-3 (1:1,000, Cat. No: 25128-1-AP), Caspase-3 (1:1,000, Cat. No: 19677-1-AP), Bcl-2 (1: 1,500, Cat. No: 26593-1-AP), GAPDH (1:20,000, Cat. No: 10494-1-AP), HRP-conjugated secondary goat anti-mouse (1: 5,000, Cat. No: SA00001-1) and goat anti-rabbit (1: 5,000, Cat. No: SA00001-2) antibodies were purchased from Proteintech Group (Rosemont, IL, United States). Immunoreactive bands were detected using the Immobilon ECL substrate kit (Millipore, Merck KGaA, Germany). Images were captured using the BioSpectrum 600 Imaging System (UVP, CA, United States). Gray values were calculated using Image J 1.46r software, with GAPDH serving as a protein loading control.

### 2.8 Immunohistochemistry

Immunohistochemistry was conducted by ServiceBiotech (Wuhan, China). Specifically, kidney tissues from mice were dissected into appropriate-sized blocks and fixed by immersion in 4% paraformaldehyde for 48 h at 4°C. Following fixation, tissue blocks were rinsed in phosphate-buffered saline (PBS) for 1 h to remove residual fixative. Tissues were subsequently processed through a standard series of ethanol dehydrations, cleared in xylene, and embedded in paraffin. Sections were cut at a thickness of 4–5 μm and mounted on glass slides. For immunohistochemical analysis, mounted sections were first deparaffinized in xylene and rehydrated through a graded ethanol series to distilled water. Antigen retrieval was performed using an appropriate buffer (citrate buffer, pH 6.0) with a heating method (water bath). Endogenous peroxidase activity was quenched by incubating sections with 3% hydrogen peroxide in PBS for 10 min at room temperature. After washing in PBS, non-specific binding sites were blocked by applying 5% normal goat serum (diluted in PBS) for 30 min at room temperature. Excess blocking serum was gently removed, and sections were incubated overnight at 4°C with primary antibodies diluted in PBS containing 5% normal goat serum. Primary antibodies targeting Bax, Cleaved Caspase-3, EZH2, and Ki67 were used accordingly. The following day, slides were warmed to room temperature and washed three times in PBS for 10 min each. A biotin-conjugated secondary antibody, diluted in 5% normal goat serum, was applied to cover the tissue sections and incubated for 45 min at room temperature. After additional PBS washes, sections were incubated with avidin-biotin-peroxidase complex (ABC reagent) for 30 min at room temperature. Following another PBS wash, immunoreactivity was visualized using a 3,3′-diaminobenzidine (DAB) substrate kit. The chromogenic reaction was monitored microscopically and stopped by immersion in tap water once optimal signal-to-background contrast was achieved. Sections were then counterstained with hematoxylin for 1–2 min, differentiated in acid alcohol if necessary, and blued in tap water. Finally, slides were dehydrated through graded alcohols, cleared in xylene, and coverslipped with a permanent mounting medium. Stained sections were examined and imaged using a bright-field microscope, and digital images were archived for analysis. All incubations were performed in a humidified chamber to prevent drying of tissues.

Images were captured using an Olympus FSX100 microscope (Olympus, Japan). Protein expression levels were analyzed by calculating the integrated optical density per stained area (IOD/area) using ImageJ 1.46r software.

### 2.9 Real-time polymerase chain reaction (RT-PCR)

Total RNA was isolated using the TRIzol Kit (ThermoFisher, Guangzhou, China). Complementary DNA (cDNA) was synthesized using the TaqMan Reverse Transcription Kit (ThermoFisher) under the following thermal cycling conditions: 95°C for 15 s, 60°C for 30 s, and 72°C for 45 s, repeated for 50 cycles. Gene expression was quantified using the 2^–ΔΔCt^ method, with primer sequences detailed in Supplementary Table 1.

### 2.10 Terminal dexynucleotidyl transferase (TdT)-mediated dUTP nick-end labeling (TUNEL) assay

Apoptotic cell levels in renal tissue sections (5 μm) were assessed using the TUNEL Apoptosis Assay Kit (Solarbio). Sections were randomly observed at 400 × magnification to identify apoptotic nuclei, indicated by yellow-brown or dark brown staining. The apoptotic index (AI) was calculated as follows: (number of apoptotic cells/total cell count) × 100%.

### 2.11 MTT assay

Cell viability was evaluated using an MTT colorimetric assay. Briefly, cells at the logarithmic phase, following different transfections, were seeded into a 96-well plate. Twenty-four hours post-transfection, 10 μL of MTT solution was added to each well, and the cells were incubated for an additional 3 h. The medium was subsequently removed, and 100 μL of dimethylsulfoxide (DMSO) was added to each well to dissolve the formazan crystals over a 10-min period. Absorbance was measured at 490 nm using a spectrophotometer (Olympus Corporation, Tokyo, Japan). The data were normalized to the untreated control group, and cell viability was calculated as a percentage.

### 2.12 Statistical analysis

Statistical analyses were performed using GraphPad Prism v8.0 (GraphPad, CA, United States). Experimental data were presented as mean ± SEM. Group comparisons were conducted using Student’s *t*-test (for two-group comparisons) or one-way ANOVA followed by Tukey’s *post-hoc* test (for multiple-group comparisons), with *p*-values < 0.05 considered statistically significant. *In vitro* experiments were conducted in triplicate (*n* = 3), while *in vivo* experiments were performed using six biological replicates per group (*n* = 6).

## 3 Results

### 3.1 MiR-144-3p expression is downregulated in renal IRI in mouse

**FIGURE 1:**
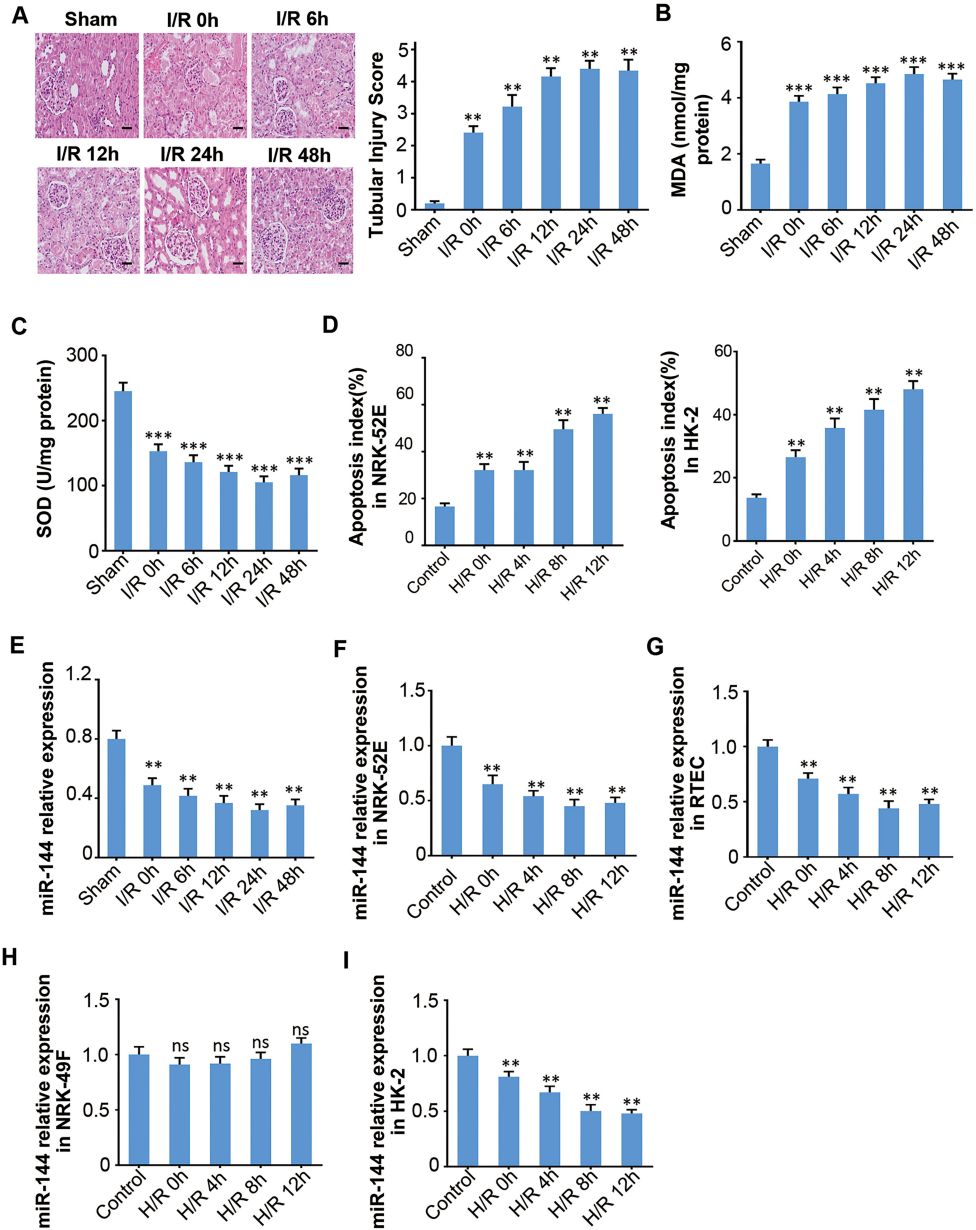
I/R and H/R induce apoptosis and suppress miR-144-3p expression in ischemic and hypoxic renal tubular cells and mouse tissues. **(A)** Representative H&E staining of mouse renal tissues at 0, 6, 12, 24, and 48 h following renal ischemia-reperfusion (I/R) injury (30 min bilateral renal pedicle clamping followed by reperfusion for the indicated durations), and tubular injury scores. Scale bar, 10 μm. **(B)** MDA levels in renal tissues from sham-operated mice and mice subjected to I/R (0–48 h). **(C)** SOD activity in renal tissues from sham-operated mice and mice subjected to I/R (0–48 h). **(D)** Representative flow cytometry dot plots and quantification of apoptosis (Annexin V-FITC/PI staining) in NRK-52E and HK-2 cells subjected to hypoxia/reoxygenation [H/R; 24 h hypoxia (94% N_2_, 5% CO_2_, 1% O_2_) followed by 4 h reoxygenation (95% air, 5% CO_2_)]. The lower right (Q3) and upper right (Q2) quadrants represent early and late apoptotic cells, respectively; their combined percentage reflects the total apoptotic population. **(E)** qRT-PCR analysis of miR-144-3p expression in mouse renal tissues after I/R. **(F)** qRT-PCR analysis of miR-144-3p expression in NRK-52E cells after H/R. **(G)** qRT-PCR analysis of miR-144-3p expression in rat renal tubular epithelial cells (RTECs) after H/R. **(H)** qRT-PCR analysis of miR-144-3p expression in rat renal fibroblast NRK-49F cells after H/R. **(I)** qRT-PCR analysis of miR-144-3p expression in human renal proximal tubular epithelial HK-2 cells after H/R. Data are presented as mean ± SEM. *In vitro* experiments were performed in triplicate (*n* = 3 independent experiments per group); *in vivo* experiments used *n* = 6 mice per group. Statistical comparisons between two groups were performed using Student’s *t*-test; comparisons among multiple groups were performed using one-way ANOVA followed by Tukey’s *post-hoc* test. A *p*-value < 0.05 was considered statistically significant. **p < 0.01, ***p < 0.001; ns, not significant (*p* > 0.05) versus the indicated control (sham or control).

To elucidate the pathogenesis of renal IRI, C57 mice and the NRK-52E cell line were utilized to establish renal I/R and H/R models, respectively. Hematoxylin-eosin (HE) staining revealed a significantly higher degree of cellular swelling and vacuolation following renal I/R injury compared to the sham group (*p* < 0.01) (Figure 1A). Oxidative stress and antioxidant capacity were assessed using malondialdehyde (MDA) and superoxide dismutase (SOD) levels, two widely recognized biochemical markers. As anticipated, MDA levels were markedly higher in the renal I/R group compared to the sham group (*p* < 0.001) (Figure 1B). On the other hand, SOD levels were significantly lower in the renal I/R group compared to the sham group (*p* < 0.001) (Figure 1C). Furthermore, flow cytometry analysis demonstrated a substantial increase in apoptosis indices following H/R treatment compared to the control group both in NRK-52E and HK-2 cellines (*p* < 0.01) (Figure 1D and Supplementary Figure 1A). Notably, miR-144-3p expression was significantly downregulated in both renal I/R-injured tissue (*p* < 0.01) (Figure 1E) and H/R-treated NRK-52E cells (*p* < 0.01) (Figure 1F) compared to their respective sham controls. To clarify the cell-type-specific and species-specific expression of miR-144-3p before and after renal IRI, we examined its levels in rat renal tubular epithelial cells (RTECs, NRK-52E), rat renal fibroblasts (NRK-49F) and Human renal tubular epithelial cell (HK-2). The results showed that miR-144-3p expression was significantly downregulated in H/R-treated RTECs, NRK-52E and HK-2, whereas no significant change was observed in H/R-treated NRK-49F cells compared with controls (Figures 1G–I).

### 3.2 MiR-144-3p inhibits cell apoptosis and promotes cell proliferation *in vitro*

**FIGURE 2:**
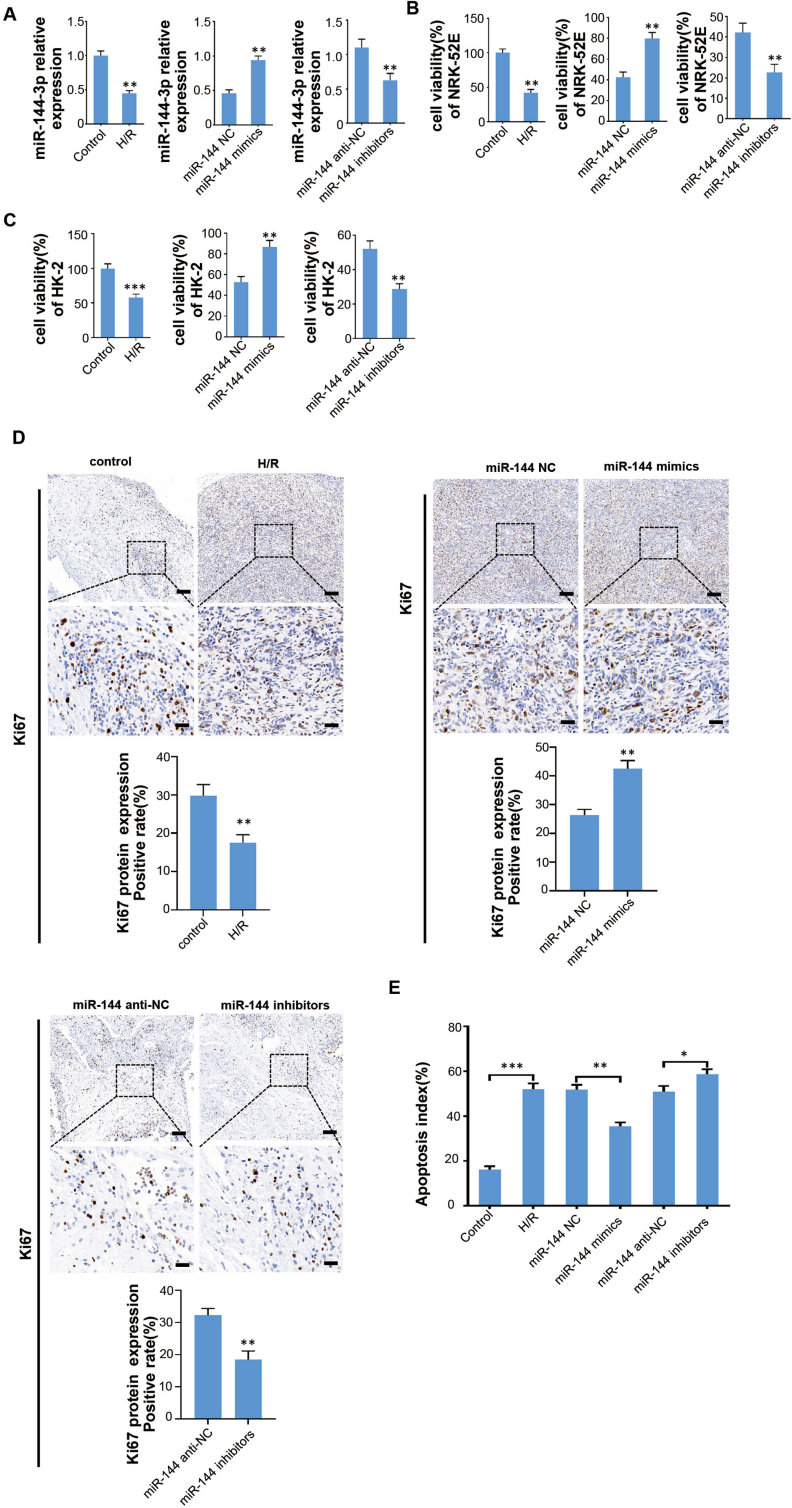
MiR-144-3p overexpression enhances cell viability in NRK-52E and HK-2 cells and promotes cell proliferation in NRK-52E cells under H/R conditions. **(A)** qRT-PCR validation of miR-144-3p overexpression and knockdown efficiency in NRK-52E cells transfected with miR-144-3p mimics, miR-144-3p inhibitors, or their respective negative controls (miR-144 NC; miR-144 anti-NC) using Lipofectamine 2000, under H/R conditions. **(B)** MTT assay showing cell viability of NRK-52E cells transfected with miR-144-3p mimics, inhibitors, or respective controls under H/R conditions **(C)** MTT assay showing cell viability of HK-2 cells transfected with miR-144-3p mimics, inhibitors, or respective controls under H/R conditions. **(D)** Representative Ki67 immunohistochemistry (IHC) images and quantification of cell proliferation in NRK-52E cells transfected as indicated, under H/R conditions. Scale bars, 20 and 50μm. **(E)** Quantification of apoptosis (Annexin V-FITC/PI staining) in NRK-52E cells transfected with miR-144-3p mimics, inhibitors, or respective controls under H/R conditions. Data indicate mean ± SEM, *n* = 3. Statistical comparisons between two groups were performed using Student’s *t*-test; comparisons among multiple groups were performed using one-way ANOVA followed by Tukey’s *post-hoc* test. *p < 0.05, **p < 0.01, ***p < 0.001 versus the indicated control.

To elucidate the role of miR-144-3p in renal IRI, NRK-52E cells were transfected with miR-144-3p mimics and inhibitors under H/R conditions (Figure 2A). MTT assays revealed that the cell viability was significantly lower in the H/R group compared to controls (*p* < 0.01). And miR-144-3p overexpression enhanced NRK-52E and HK-2 cell viability, whereas miR-144-3p knockdown reduced viability under H/R conditions (*p* < 0.01) (Figures 2B,C). To evaluate the effect of miR-144-3p on cell proliferation under H/R conditions, Ki67 immunohistochemistry (IHC) was performed. The results showed that Ki67 expression was significantly reduced in the H/R group compared with controls. Overexpression of miR-144-3p markedly increased Ki67 levels, whereas miR-144-3p knockdown further suppressed cell proliferation under H/R conditions (*p* < 0.01) (Figure 2D).

**FIGURE 3:**
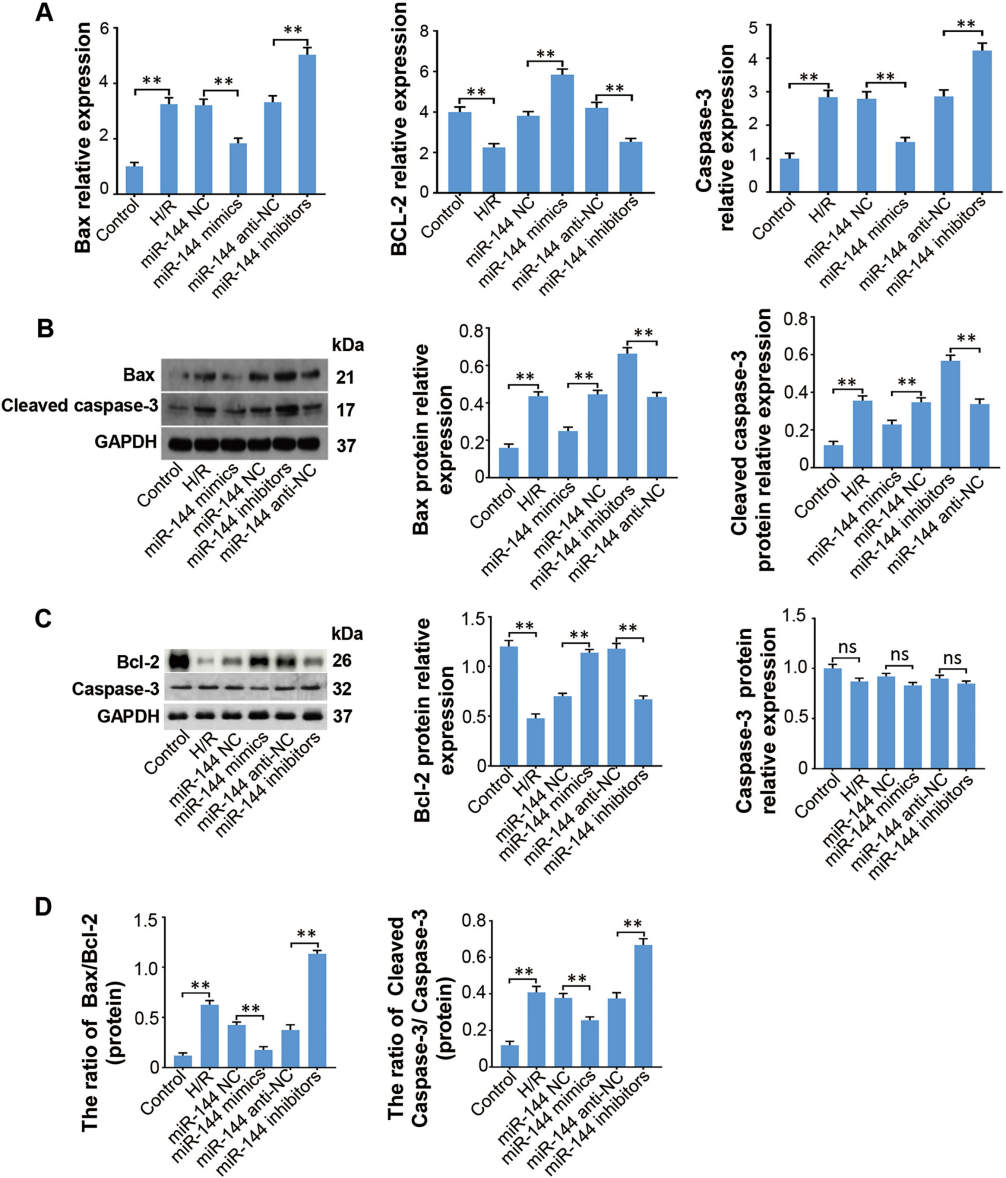
MiR-144-3p modulates the expression of apoptosis-related genes in NRK-52E cells under H/R conditions. **(A)** qRT-PCR analysis of Bax, Bcl-2, and Caspase-3 mRNA expression in NRK-52E cells transfected with miR-144-3p mimics, inhibitors, or respective controls under H/R conditions. **(B,C)** Representative western blot images of Bax, Bcl-2, cleaved Caspase-3, and Caspase-3 protein expression in NRK-52E cells treated as in **(A)**. GAPDH served as a loading control. Densitometric quantification of Bax, Bcl-2, Caspase-3, and cleaved Caspase-3 protein levels (normalized to GAPDH) were statistical analysis. **(D)** Quantification of the Bax/Bcl-2 protein ratio and the cleaved Caspase-3/Caspase-3 ratio, calculated from the densitometric data in **(B,C)**. Data indicate mean ± SEM, *n* = 3. Statistical comparisons between two groups were performed using Student’s *t*-test; comparisons among multiple groups were performed using one-way ANOVA followed by Tukey’s *post-hoc* test. **p < 0.01; ns, not significant (*p* > 0.05) versus the indicated control [control, miR-144-3p negative control (miR-144 NC), anti-miR-144-3p negative control (miR-144 anti-NC)].

To further investigate the impact of miR-144-3p on apoptosis, flow cytometry analysis was performed. The results demonstrated that miR-144-3p overexpression significantly inhibited apoptosis (*p* < 0.01), while miR-144-3p knockdown promoted apoptosis in NRK-52E cells subjected to H/R injury (*p* < 0.05) (Figure 2E and Supplementary Figure 1B). Mechanistic studies using qRT-PCR and western blot analyses revealed that miR-144-3p modulates apoptotic signaling in renal tubular epithelial cells. At the mRNA level, miR-144-3p overexpression significantly downregulated the pro-apoptotic marker *Bax* and upregulated the anti-apoptotic marker Bcl-2 (*p* < 0.01) (Figure 3A). At the protein level, miR-144-3p similarly decreased Bax and cleaved Caspase-3 expression while increasing Bcl-2 levels (*p* < 0.01) (Figures 3B,C). In contrast, inhibition of miR-144-3p resulted in upregulation of Bax and downregulation of Bcl-2 (*p* < 0.01) (Figures 3B,C). Notably, although miR-144-3p reduced *Caspase-3* mRNA expression (*p* < 0.01) (Figure 3A), no significant change was observed at the protein level (*p* > 0.05) (Figure 3C). To further quantify the effect on apoptosis, the Bax/Bcl-2 protein ratio and the cleaved Caspase-3/Caspase-3 ratio were analyzed. The results showed that miR-144-3p decreased both ratios, whereas inhibition of miR-144-3p produced the opposite effect (Figure 3D), confirming its anti-apoptotic role.

### 3.3 EZH2 facilitates cell apoptosis during IRI events

**FIGURE 4:**
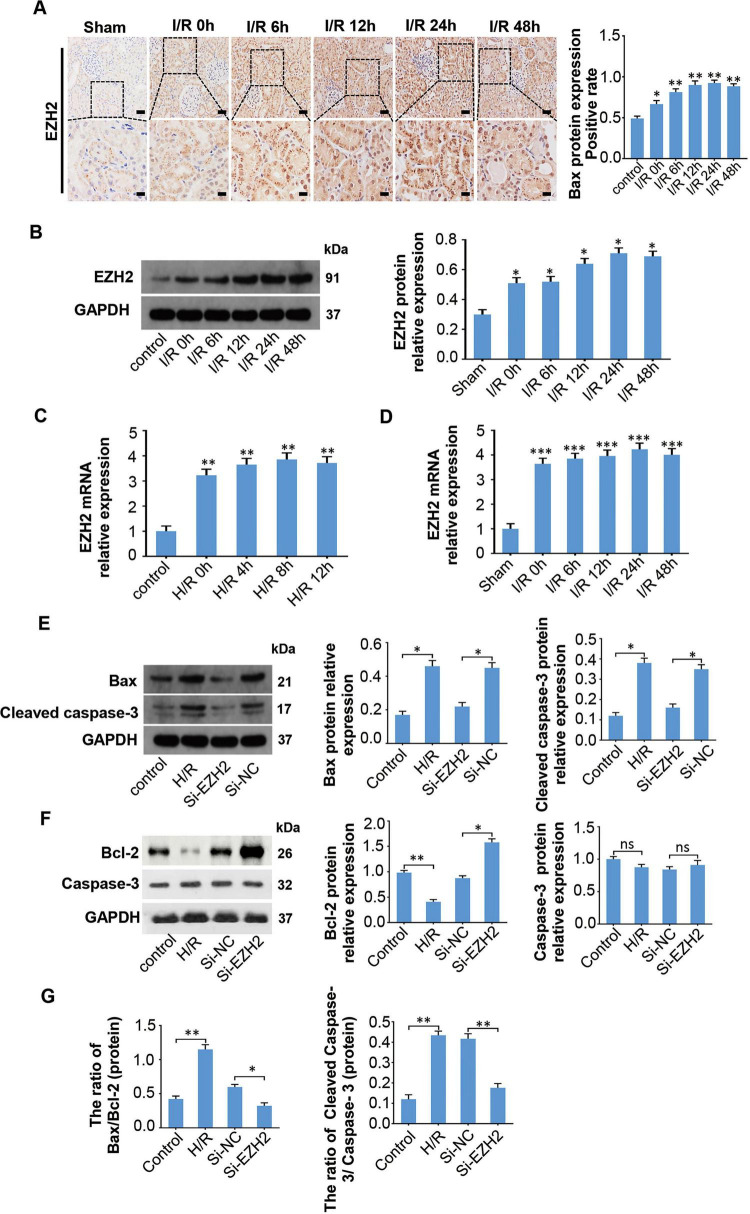
EZH2 is downregulated in I/R-treated mouse tissues and renal tubular cells under H/R conditions, and its inhibition reduces Bax and Caspase-3 expression in NRK-52E cells under H/R conditions. **(A)** Representative IHC images of EZH2 protein expression in renal tissues from sham and I/R-treated mice. Scale bars, 20 and 50 μm. **(B)** Western blot analysis and densitometric quantification of EZH2 protein levels in renal tissues from sham and I/R-treated mice. **(C)** qRT-PCR analysis of EZH2 mRNA expression in NRK-52E cells under H/R conditions. **(D)** qRT-PCR analysis of EZH2 mRNA expression in mouse renal tissues after I/R. **(E,F)** Representative western blot images of Bax, Bcl-2, Caspase and cleaved Caspase-3 protein expression in NRK-52E cells transfected with EZH2-specific siRNA (si-EZH2) or negative control siRNA (si-NC), under H/R conditions. Densitometric quantification of Bax, Bcl-2 Capsese 3 and Caspase-3 protein expression in NRK-52E cells (normalized to GAPDH) were statistical analysis. **(G)** Quantification of the Bax/Bcl-2 protein ratio and the cleaved Caspase-3/Caspase-3 ratio, calculated from densitometric data in **(E,F)**. Data indicate mean ± SEM (*n* = 3 for cell-based assays; *n* = 6 for animal studies). Statistical comparisons between two groups were performed using Student’s *t*-test; comparisons among multiple groups were performed using one-way ANOVA followed by Tukey’s *post-hoc* test. A *p*-value < 0.05 was considered statistically significant. *p < 0.05, **p < 0.01, ***p < 0.001; ns, not significant (*p* > 0.05) versus the indicated control.

In this study, immunohistochemistry revealed that the expression level of EZH2 was significantly higher in I/R mice compared to the control group (Figure 4A). Likewise, Western blot analysis indicated that EZH2 protein levels were higher in the I/R group compared to the control group (*p* < 0.05) (Figure 4B). To further assess *EZH2* expression at the mRNA level, PCR analysis was conducted both *in vivo* and *in vitro*. The results indicated that *EZH2* mRNA expression levels were markedly higher in the I/R group (*p* < 0.001), as well as in NRK-52E cells exposed to H/R conditions (*p* < 0.01), compared to their respective controls (Figures 4C,D). Taken together, these findings suggest that inhibiting EZH2 protein expression may confer protective effects against renal IRI. Additionally, western blot analysis further demonstrated the effect of *EZH2* on apoptosis. The pro-apoptotic proteins Bax and cleaved Caspase-3 were significantly upregulated in the H/R groups, whereas their expression was markedly reduced in si-*EZH2*-transfected H/R cells compared with respective controls (*p* < 0.05) (Figure 4E). In contrast, the anti-apoptotic protein Bcl-2 was downregulated in the H/R groups and upregulated following *EZH2* knockdown (*p* < 0.05) (Figure 4F). Caspase-3 levels, however, did not show significant changes under the same conditions (Figure 4F). To further quantify the apoptotic effect of EZH2, the Bax/Bcl-2 protein ratio and the cleaved Caspase-3/Caspase-3 ratio were calculated. Both ratios were decreased in si-*EZH2*-transfected cells (Figure 4G), confirming the pro-apoptotic role of *EZH2*.

### 3.4 MiR-144-3p inhibits the expression of EZH2, Bax, and Caspase-3 *in vitro*

**FIGURE 5:**
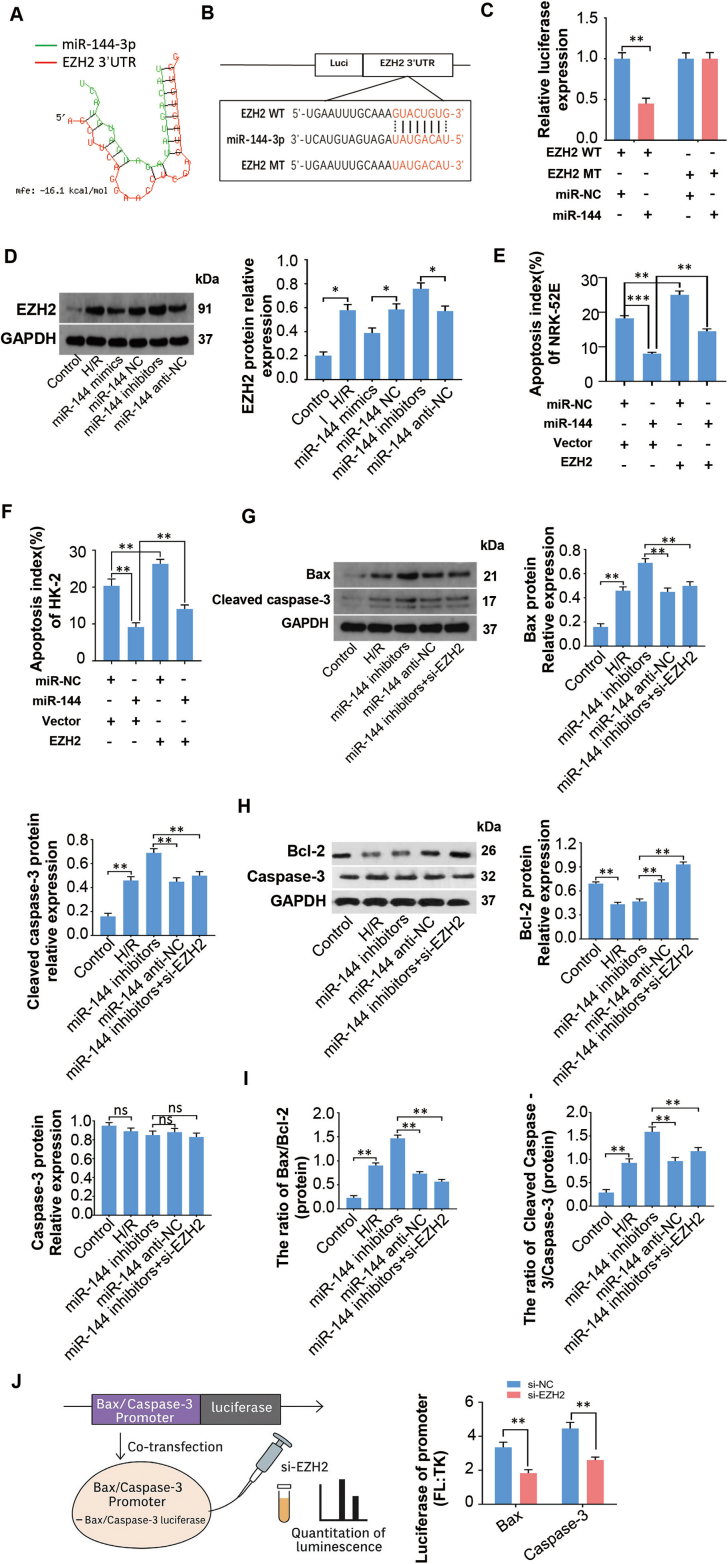
MiR-144-3p suppresses EZH2 expression by targeting the 3′UTR of EZH2, and EZH2 inhibition reduces Bax and Caspase-3 expression in NRK-52E cells under H/R conditions. **(A)** The predicted miR-144-3p binding site of EZH2 3′UTR by RNAhybrid 2.2. **(B)** Sequence alignment showing the wild-type (EZH2-WT) and mutant (EZH2-MT) miR-144-3p binding sites in the EZH2 3′UTR, and the mature miR-144-3p sequence. **(C)** Dual-luciferase reporter assay in NRK-52E cells co-transfected with miR-144-3p mimics or negative control (miR-NC) and luciferase reporter constructs containing either EZH2-WT or EZH2-MT 3′UTR. Firefly luciferase activity was normalized to Renilla luciferase activity. **(D)** Western blot analysis and densitometric quantification of EZH2 protein expression in NRK-52E cells transfected with miR-144-3p mimics, inhibitors, or respective controls, under H/R conditions. **(E,F)** Quantification of flow cytometry apoptosis in NRK-52E and HK-2 cells co-transfected with miR-144-3p mimics (or miR-NC) and EZH2 overexpression plasmid (or empty vector), under H/R conditions. **(G,H)** Representative western blot images of Bax, Bcl-2, Caspase 3 and cleaved Caspase-3 protein expression in NRK-52E cells transfected with miR-144-3p inhibitors alone or co-transfected with si-EZH2, under H/R conditions, and densitometric quantification of Bax, Bcl-2, Caspase 3 and cleaved Caspase-3 protein expression in NRK-52E cells (normalized to GAPDH) were statistical analysis. **(I)** The Bax/Bcl-2 protein ratio and the cleaved Caspase-3/Caspase-3 ratio were analyzed from **(G,H)**. **(J)** Dual-luciferase reporter assay of Bax and Caspase-3 promoter activity in NRK-52E cells co-transfected with promoter-driven luciferase constructs (pGL3-Bax or pGL3-Caspase-3) and either si-EZH2 or si-NC, under H/R conditions. Data indicate mean ± SEM, *n* = 3. Statistical comparisons between two groups were performed using Student’s *t*-test; comparisons among multiple groups were performed using one-way ANOVA followed by Tukey’s *post-hoc* test. *p < 0.05, **p < 0.01, ***p < 0.001; ns, not significant (*p* > 0.05) versus the indicated control.

To investigate the mechanisms underlying the downregulation of *EZH2* in renal IRI, a dual-luciferase reporter assay was performed. The interaction between miR-144-3p and the *EZH2* 3′UTR was predicted using RegRNA 2.2 (Figure 5A). Wild-type (WT) and mutant (MT) sequences of the *EZH2* 3′UTR were designed and cloned into the pmirGLO vector for transfection (Figure 5B). The results implied that relative luciferase activity was significantly lower in cells co-transfected with the miR-144-3p mimics and *EZH2* 3′UTR-WT (*p* < 0.01), whereas no significant change was observed in cells co-transfected with the miR-144-3p mimics and *EZH2* 3′UTR-MT in NRK-52E cells (*p* > 0.05) (Figure 5C). These results collectively demonstrate that miR-144-3p directly binds to the 3′UTR of *EZH2*.

To elucidate the regulatory mechanism of miR-144-3p on *EZH2* in renal IRI, EZH2 protein expression levels were examined by modulating miR-144-3p levels in NRK-52E cells under H/R conditions. Western blot analysis signaled that EZH2 protein levels were higher in both the H/R group and cells transfected with the miR-144-3p mimics compared to their respective controls. In comparison, transfection with miR-144-3p inhibitors significantly reduced EZH2 protein levels in NRK-52E cells (*p* < 0.05) (Figure 5D). To further corroborate that *EZH2* mediates the anti-apoptotic effect of miR-144-3p, a rescue experiment was conducted by co-transfecting NRK-52E and HK-2 cells with miR-144-3p mimics and an *EZH2* overexpression plasmid lacking the 3′ UTR. Flow cytometry analysis showed that miR-144-3p significantly attenuated apoptosis (*p* < 0.001), whereas *EZH2* overexpression partially reversed this effect, leading to a moderate increase in the proportion of apoptotic cells compared to miR-144-3p alone both in NRK-52E and HK-2 (*p* < 0.01) (Figures 5E,F and Supplementary Figure 1C).

To further explore the interplay between miR-144-3p, *EZH2*, and downstream apoptotic proteins such as Bax and Caspase-3, reversal experiments were carried out using miR-144-3p inhibitors and *EZH2*-specific siRNA (si-*EZH2*). The results demonstrated that miR-144-3p inhibitors upregulated Bax and cleaved Caspase-3 protein expression, whereas si-*EZH2* reversed the pro-apoptotic effects of miR-144-3p inhibition, leading to decreased Bax and cleaved Caspase-3 expression in NRK-52E cells under H/R conditions (*p* < 0.01) (Figure 5G). Bcl-2 expression was increased following transfection with miR-144-3p inhibitors, whereas co-transfection with si-*EZH2* reversed this effect, mitigating the pro-apoptotic impact of miR-144-3p inhibition (*p* < 0.01) (Figure 5H). Caspase-3 levels did not show significant changes under the same conditions (*p* > 0.05) (Figure 5H). To further assess apoptosis, the Bax/Bcl-2 protein ratio and the cleaved Caspase-3/Caspase-3 ratio were calculated. Both ratios were elevated in cells transfected with miR-144-3p inhibitors, and co-transfection with si-*EZH2* reversed these effects, confirming the regulatory role of the miR-144-3p/*EZH2* axis in apoptosis (*p* < 0.01) (Figure 5I).

To investigate the regulatory effects of *EZH2* on the expression of Bax and cleaved Caspase-3, luciferase reporter constructs containing their promoter regions were co-transfected with si-*EZH2*. Interestingly, *EZH2* knockdown significantly decreased luciferase activity driven by both the *Bax* and *Caspase-3* promoters (*p* < 0.01), indicating that *EZH2* positively regulates the transcriptional activity of these pro-apoptotic genes in the context of H/R-treated NRK-52E cells. Given *EZH2*’s canonical role as a transcriptional repressor through PRC2-mediated H3K27me3 deposition, this result suggests either an indirect regulatory mechanism (e.g., *EZH2*-mediated silencing of a transcriptional repressor of *Bax*/*Caspase-3*) or a non-canonical co-activator function of *EZH2* in this experimental context (Figure 5J). The potential mechanisms underlying this observation are further addressed in the Discussion.

### 3.5 MiR-144-3p inhibits cell apoptosis during renal IRI *in vivo*

**FIGURE 6:**
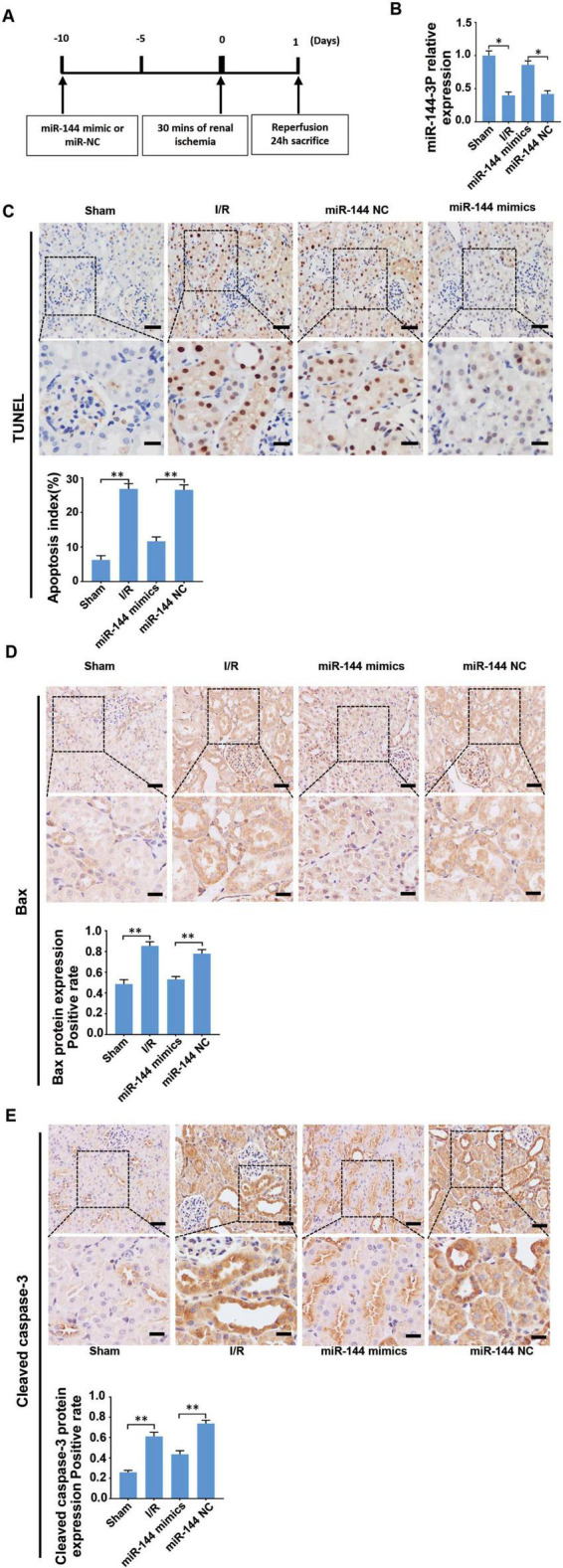
MiR-144-3p attenuates apoptosis in a mouse model of renal IRI *in vivo*. **(A)** Schematic of the experimental timeline: C57BL/6 male mice (8–10 weeks, 20–25 g) received tail-vein injections of miR-144-3p agomir or negative control (miR-NC) once daily for 10 consecutive days, followed by bilateral renal ischemia (30 min clamping) and reperfusion (24 h). **(B)** qRT-PCR analysis of miR-144-3p expression in renal tissues from sham, I/R, miR-144-3p agomir + I/R, and miR-NC + I/R groups. **(C)** Representative TUNEL staining images and quantification of apoptotic cells in renal tissue sections from the indicated groups. Scale bars, 20 and 50 μm. **(D,E)** Representative IHC images and quantification of Bax and cleaved Caspase-3 protein expression in renal tissue sections from its indicated groups. Scale bars, 20 and 50 μm. Data indicate mean ± SEM (*n* = 3 for cell-based assays; *n* = 6 for animal studies). Statistical comparisons were performed using Student’s *t*-test (two-group) or one-way ANOVA followed by Tukey’s *post-hoc* test (multiple groups). *p < 0.05, **p < 0.01 versus the indicated control.

To further validate the protective role of miR-144-3p in suppressing cell apoptosis during renal IRI *in vivo*, mice were injected with miR-144-3p mimics and negative oligo (miR-NC) for 10 days, followed by 30 min of bilateral renal ischemia and 24 h of reperfusion (Figure 6A). Quantitative RT-PCR analysis unveiled that miR-144-3p expression levels were significantly lower in the I/R group compared to the sham group (*p* < 0.05), whereas they were 2-fold higher in the miR-144-3p-transfected group compared to its controls (Figure 6B). To further elucidate the biological function of miR-144-3p in IRI, a TUNEL assay was performed, and the results demonstrated a marked reduction in the number of apoptotic nuclei in miR-144-3p-treated mice compared to controls (*p* < 0.05) (Figure 6C).

**FIGURE 7:**
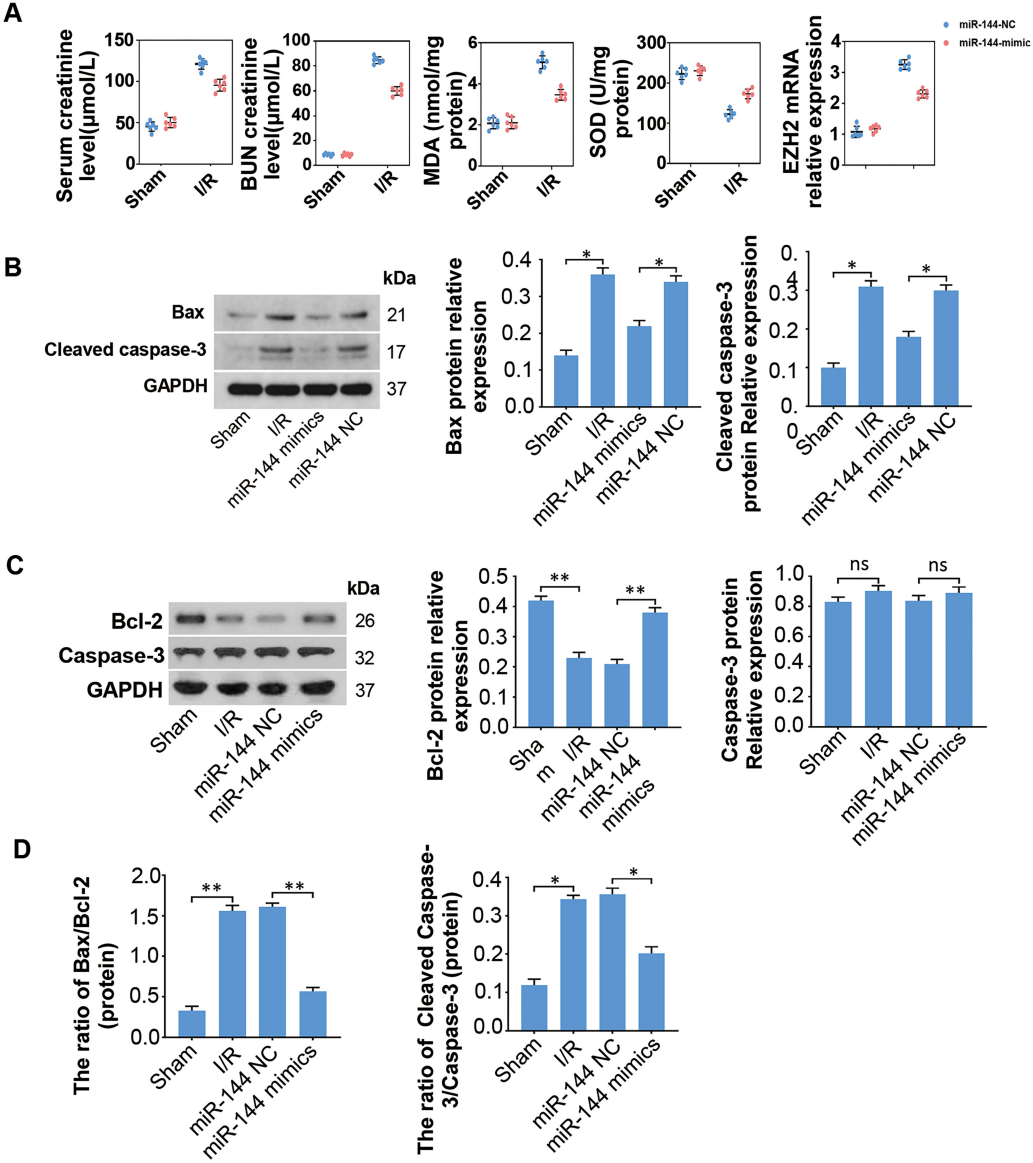
MiR-144-3p preserves renal function and inhibits EZH2-mediated apoptosis in renal IRI *in vivo* **(A)**. **(A)** Serum creatinine, blood urea nitrogen (BUN), renal MDA, renal SOD, and renal EZH2 mRNA levels in sham, I/R, miR-144-3p agomir + I/R, and miR-NC + I/R groups. Measurements were performed after the treatment regimen described in Figure 6A. **(B,C)** Representative western blot images of Bax, Bcl-2, cleaved Caspase-3, and Caspase-3 protein expression in renal tissues from the indicated groups. GAPDH served as a loading control. Densitometric quantification of Bax, Bcl-2, cleaved Caspase-3, and Caspase-3 protein levels (normalized to GAPDH) were statistical analysis. **(D)** Quantification of the Bax/Bcl-2 protein ratio and the cleaved Caspase-3/Caspase-3 ratio, calculated from the densitometric data in **(C)**. Data indicate mean ± SEM (*n* = 3 for cell-based assays; *n* = 6 for animal studies). Statistical comparisons were performed using Student’s *t*-test (two-group) or one-way ANOVA followed by Tukey’s *post-hoc* test (multiple groups). *p < 0.05, **p < 0.01; ns, not significant (*p* > 0.05) versus the indicated control.

To investigate the regulatory effects of miR-144-3p on pro-apoptotic proteins Bax and cleaved Caspase-3, immunohistochemistry was conducted. The results showed that Bax and cleaved Caspase-3 expression levels were upregulated in I/R mice but significantly downregulated in miR-144-3p-treated mice compared to their respective controls (Figures 6D,E). To evaluate the protective effect of miR-144-3p on renal function and its regulation of *EZH2*, serum creatinine, blood urea nitrogen (BUN), MDA, SOD, and *EZH2* mRNA levels were measured before and after administration of miR-144-3p mimics. The results demonstrated that miR-144-3p mimics significantly reduced *EZH2* mRNA expression, decreased serum creatinine and BUN levels, inhibited MDA accumulation, and enhanced SOD activity (Figure 7A). These findings indicate that miR-144-3p mimics effectively protect renal function and mitigate oxidative stress in the context of ischemia-reperfusion injury. Western blot analysis further confirmed these findings. The results showed that Bax and cleaved Caspase-3 were upregulated in I/R-treated tissues, whereas their expression was markedly reduced in the miR-144-3p mimic groups compared with respective controls. Caspase-3 levels did not show significant changes between I/R or miR-144-3p-treated groups and their corresponding sham or control groups (*p* < 0.05) (Figures 7B,C). To further quantify apoptosis, the Bax/Bcl-2 protein ratio and the cleaved Caspase-3/Caspase-3 ratio were calculated, both of which were decreased in cells transfected with miR-144-3p mimics (*p* < 0.05) (Figure 7D), supporting the anti-apoptotic effect of miR-144-3p.

**FIGURE 8:**
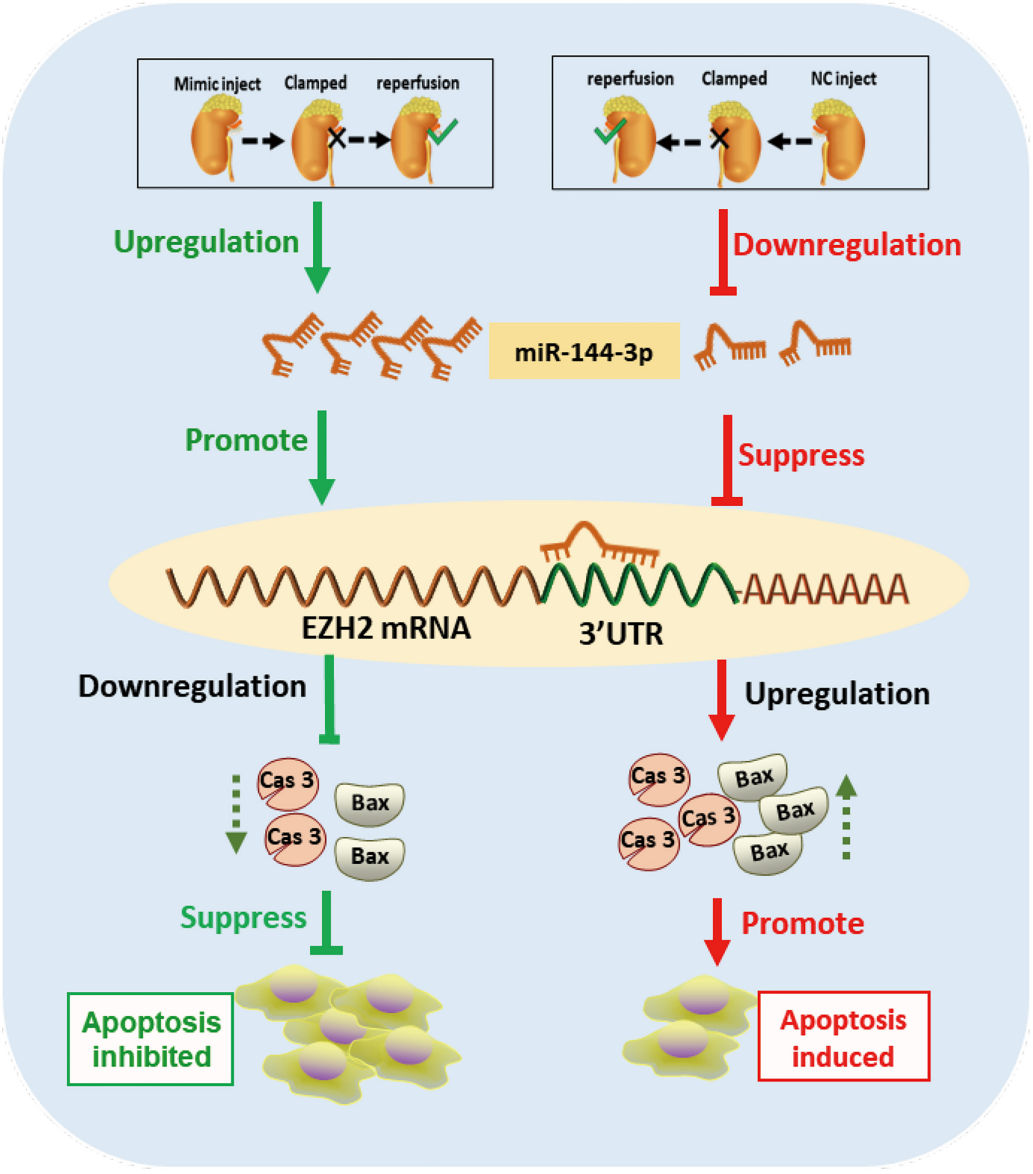
Schematic diagram illustrating the proposed miR-144-3p/EZH2 regulatory pathway in renal ischemia-reperfusion injury. Under I/R or H/R conditions, miR-144-3p expression is downregulated, leading to the upregulation of EZH2. EZH2 promotes the expression of the pro-apoptotic proteins Bax and cleaved Caspase-3, thereby facilitating renal tubular epithelial cell apoptosis. miR-144-3p overexpression suppresses EZH2 expression through direct binding to its 3′UTR, reducing Bax and cleaved Caspase-3 levels and attenuating apoptosis.

Collectively, these results suggest that miR-144-3p exerts a negative regulatory effect on *EZH2* through a post-transcriptional mechanism, leading to the downregulation of *Bax* and cleaved Caspase-3, thereby inhibiting cell apoptosis in an *in vitro* model of renal ischemia-reperfusion injury, as illustrated in the schematic representation of the mechanism of action (Figure 8).

## 4 Discussion

This study demonstrated that miR-144-3p expression was significantly reduced in rat renal epithelial NRK-52E cells under H/R conditions and in mouse renal tissues subjected to IRI. Overexpression of miR-144-3p significantly attenuated cell apoptosis and enhanced cell proliferation, whereas inhibition of miR-144-3p exacerbated cell apoptosis under IRI or H/R injury conditions. Further investigation revealed that miR-144-3p negatively regulates *EZH2* via post-transcriptional mechanisms, while *EZH2* mediates the protective effects of miR-144-3p through a negative feedback pathway in renal IRI. Of note, mechanistic studies showed that miR-144-3p targets the *EZH2* 3′UTR, and *EZH2* promotes cell apoptosis by upregulating the Bax/cleaved Caspase-3 pathway. Overall, these findings suggest that miR-144-3p plays a protective role in renal IRI by negatively regulating *EZH2* and, subsequently, Bax and cleaved Caspase-3 expression. These insights position miR-144-3p as a potential therapeutic target for the treatment of acute kidney injury (AKI) and for improving outcomes in renal transplantation.

Accumulating evidence indicates that renal ischemia-reperfusion (I/R) injury is a leading cause of acute kidney failure and renal damage (25, 26). While reperfusion is essential to prevent irreversible ischemic damage, it paradoxically initiates oxidative stress and inflammatory responses that exacerbate injury (27, 28). Although the underlying mechanisms remain incompletely understood, emerging studies have identified microRNAs (miRNAs) as pivotal post-transcriptional regulators involved in the pathogenesis of I/R injury across various organs (28, 29). Among them, miR-144-3p has been implicated in diverse biological processes and diseases, functioning in a context-dependent manner. For instance, it has been shown to act as a tumor suppressor in osteosarcoma by inducing ferroptosis through ZEB1 inhibition (19). Conversely, miR-144-3p has also been reported to promote tumor progression in hepatocellular carcinoma by targeting EIF4G2 and activating the ERK pathway (29), highlighting its dual role in oncogenesis. In I/R injury, miR-144-3p exhibits tissue-specific regulatory effects. In myocardial I/R injury, miR-144-3p exerts a protective effect by directly targeting and suppressing the calcium-activated chloride channel TMEM16A (ANO1), thereby inhibiting the subsequent activation of the NLRP3 inflammasome (30). In the context of renal I/R injury, a competing endogenous RNA (ceRNA) mechanism has been identified, whereby the long non-coding RNA TUG1 sponges miR-144-3p to alleviate oxidative stress and apoptosis by upregulating Nrf2 (20). Our current study complements this upstream regulatory axis by demonstrating that miR-144-3p itself directly targets *EZH2* to inhibit apoptosis in renal IRI, further underscoring the multifaceted role of this miRNA in renal pathophysiology. Conversely, in cerebral I/R injury, evidence suggests miR-144-3p may function differently; lncRNA Rian attenuates apoptosis by acting as a ceRNA for miR-144-3p, leading to the upregulation of GATA3 (31). This regulatory pattern implies a potential injury-promoting role for miR-144-3p in the brain, which contrasts with its protective function observed in our renal IRI model and highlights the context-dependent nature of its biological actions. Noteworthily, other miRNAs also play significant roles in renal I/R injury. For example, an earlier study pointed out that miR-10a exacerbates renal I/R injury by downregulating PIK3CA expression (15), while miR-132-3p aggravates renal I/R injury by targeting the Sirt1/PGC1alpha axis (14). In contrast, miR-29a-3p attenuates renal ischemic injury by targeting TNFR1 and collagen I (16). Additionally, miR-24 has been implicated in promoting renal ischemic injury by stimulating apoptosis in endothelial and tubular epithelial cells (10). These findings highlight the intricate and context-dependent roles of miRNAs in regulating the balance between injury and repair during renal I/R injury.

Herein, miR-144-3p expression was significantly downregulated in renal I/R tissues and hypoxia/reoxygenation-treated NRK-52E cells. Functional experiments demonstrated that miR-144-3p mimics enhanced cell viability, inhibited apoptosis, and alleviated tubular injury and inflammation, supporting its protective role in renal I/R injury. Our findings demonstrate that miR-144-3p intervention markedly alleviated renal ischemia–reperfusion (I/R)–induced histopathological injury, as evidenced by reduced tubular necrosis and interstitial edema. These morphological improvements strongly suggest a mitigation of the inflammatory response. It is well established that the pathogenesis of renal ischemia–reperfusion injury (IRI) involves substantial infiltration of inflammatory cells, particularly neutrophils and macrophages, which directly contribute to tubular damage (32, 33). Such cellular recruitment is typically initiated and sustained by a cascade of pro-inflammatory mediators (34). Therefore, the pronounced reduction in histological injury observed in the miR-144-3p treatment group likely reflects a downstream suppression of inflammatory cell recruitment and activity. Future studies incorporating immunohistochemical or immunofluorescent staining for specific leukocyte markers would be warranted to further substantiate this mechanism.

While our findings are in line with the protective roles observed in cardiac and cerebral I/R models, they also emphasize the need for further investigation into the upstream regulators and broader downstream pathways of miR-144-3p, as well as its potential crosstalk with inflammation and oxidative stress responses in renal tissue. While the present study demonstrates the regulatory role of miR-144-3p in renal ischemia-reperfusion injury at the mechanistic level, its translational potential remains to be fully explored. Specifically, evaluating the therapeutic efficacy of miR-144-3p mimics administered after the onset of injury is anticipated to provide more clinically relevant insights. Future studies incorporating such intervention models are necessitated to assess its viability as a therapeutic candidate.

In addition to apoptosis and proliferation, renal IRI is characterized by robust inflammatory responses and oxidative stress (5, 6, 27). In the current study, we observed significantly elevated MDA levels and reduced SOD activity in renal tissues following I/R (Figures 1B,C), confirming the presence of oxidative stress in our model. However, the contribution of the miR-144-3p/*EZH2* axis to the regulation of inflammation and oxidative stress was not directly assessed in this study. Notably, *EZH2* has been implicated in the regulation of inflammatory responses in AKI. Li et al. demonstrated that *EZH2* promotes the expression of pro-inflammatory cytokines, including IL-6, TNF-α, IL-1β, and MCP-1, in sepsis-induced AKI, and that pharmacological inhibition of EZH2 suppresses renal inflammatory cell infiltration (35). Furthermore, the lncRNA JPX/EZH2 axis has been shown to modulate inflammatory signaling in myocardial IRI (23). Given that miR-144-3p overexpression suppresses EZH2 expression, it is plausible that the protective effects of miR-144-3p in renal IRI extend beyond apoptosis inhibition to include the attenuation of *EZH2*-mediated inflammatory responses. Additionally, miR-144-3p has been reported to regulate oxidative stress through Nrf2 targeting in renal tubular epithelial cells (20), suggesting a potential convergence of the miR-144-3p/*EZH2* axis with oxidative stress pathways. Future studies systematically evaluating inflammatory mediators (e.g., IL-6, TNF-α, IL-1β, MCP-1), tubular injury markers (e.g., KIM-1, NGAL), and additional oxidative stress parameters (e.g., ROS, GSH) in the context of miR-144-3p/*EZH2* modulation would provide a more comprehensive understanding of the protective mechanisms of miR-144-3p in renal IRI.

*EZH2* is a histone methyltransferase that primarily functions as the catalytic subunit of Polycomb Repressive Complex 2 (PRC2), catalyzing the trimethylation of histone H3 at lysine 27 (H3K27me3) to mediate transcriptional repression of target genes (36). However, accumulating evidence has revealed that *EZH2* also exerts PRC2-independent, non-canonical functions, including transcriptional co-activation, direct methylation of non-histone substrates, and protein-protein interactions with transcription factors independent of its methyltransferase activity (37, 38). For instance, *EZH2* has been shown to interact with RelA/NF-κB and function as a transcriptional co-activator independent of PRC2 in triple-negative breast cancer (38), and to cooperate with cMyc at actively transcribed gene loci devoid of H3K27me3 marks in multiple myeloma (39). In the context of renal IRI, the role of *EZH2* appears to be context-dependent. Previous studies have shown that *EZH2* can be regulated by upstream non-coding RNAs in ischemia-reperfusion injury. For instance, the lncRNA JPX directly binds to *EZH2* and attenuates its H3K27me3-mediated repression of the SERCA2a promoter, thereby protecting against myocardial IRI (23). In renal IRI, *EZH2* inhibition by 3-DZNep or GSK-126 has been reported to reduce apoptosis and inflammatory responses, partly through inactivating p38 MAPK signaling (40). More recently, *EZH2* was found to epigenetically silence SALL1 and Sox9 via H3K27me3 deposition at their promoters, thereby suppressing Wnt/β-catenin-mediated survival signaling and indirectly promoting *Bax* and *Caspase-3*-dependent apoptosis in renal tubular epithelial cells (35).

In the present study, we observed that *EZH2* knockdown significantly reduced luciferase activity driven by the *Bax* and *Caspase-3* promoters, suggesting that *EZH2* positively regulates the transcriptional activity of these pro-apoptotic genes. This finding is seemingly paradoxical to *EZH2*’s canonical role as a transcriptional repressor. We propose several potential explanations that warrant further investigation. First, *EZH2* may indirectly regulate *Bax* and *Caspase-3* transcription through a “repression-of-repressor” mechanism: *EZH2* may epigenetically silence one or more transcriptional repressors of *Bax*/*Caspase-3* (such as SALL1, Sox9, or other PRC2 targets), such that *EZH2* knockdown relieves the repression of these repressors, leading to their upregulation and subsequent suppression of *Bax* and *Caspase-3* promoter activity. This would be consistent with *EZH2*’s canonical PRC2/H3K27me3-dependent repressive function. Second, *EZH2* may exert non-canonical transcriptional co-activator functions in renal tubular epithelial cells under stress conditions. Under hypoxia/reoxygenation, post-translational modifications (e.g., AKT-mediated phosphorylation at S21, or JAK3-mediated phosphorylation at Y244) could promote EZH2 dissociation from PRC2 and enable its interaction with transcription factors such as NF-κB or STAT3 at the *Bax* and *Caspase-3* promoters, functioning as a co-activator independent of H3K27me3. Third, *EZH2* may activate p38 MAPK signaling, which has been shown to promote caspase-3 cleavage and may also enhance *Bax* transcription (40). Future studies employing chromatin immunoprecipitation followed by quantitative PCR (ChIP-qPCR) to assess H3K27me3 and *EZH2* enrichment at the *Bax* and *Caspase-3* promoters, combined with PRC2-specific inhibitors (e.g., GSK-126) versus *EZH2* degraders to dissect PRC2-dependent from PRC2-independent functions, will be essential to distinguish among these possibilities.

We acknowledge that the precise molecular mechanism by which *EZH2* positively regulates *Bax* and *Caspase-3* transcription in renal IRI remains to be fully elucidated, and this represents a limitation of the current study. Nevertheless, our functional data consistently demonstrate that *EZH2* knockdown reduces *Bax* and *Caspase-3* expression at both the mRNA and protein levels and attenuates apoptosis in NRK-52E cells under H/R conditions. *EZH2* was selected as a primary target of interest based on prior evidence of its involvement in cell fate regulation during IRI, as well as predicted binding interactions with miR-144-3p. While this study focused on the *EZH2*-mediated apoptotic pathway, we acknowledge that miR-144-3p may also regulate other injury-relevant mechanisms, such as inflammation and oxidative stress. Further studies are required to comprehensively explore these alternative downstream pathways and fully elucidate the multifaceted role of miR-144-3p in renal IRI.

*EZH2* is a histone methyltransferase that exerts its function primarily by catalyzing the trimethylation of histone H3 at lysine 27 (H3K27me3), leading to transcriptional repression of target genes (36). Previous studies have concluded that *EZH2* can be regulated by upstream non-coding RNAs and play context-dependent roles in ischemia-reperfusion injury. For instance, the long non-coding RNA JPX directly binds to *EZH2* and attenuates its H3K27me3-mediated repression of the SERCA2a promoter, thereby protecting against myocardial IRI (23). Additionally, Italiano et al. evinced that miR-322/503 upregulates *EZH2* via Smurf2 inhibition, activating the Akt/GSK3 pathway and promoting cell survival under IRI conditions (40). Herein, both *EZH2* mRNA and protein expression levels were significantly suppressed in renal I/R tissues and hypoxia/reoxygenation-treated NRK-52E cells. Knockdown of *EZH2* down-regulated the expression of pro-apoptotic proteins Bax and cleaved Caspase-3, suggesting that *EZH2* positively regulates apoptosis in renal tubular epithelial cells. *EZH2* was selected as a primary target of interest based on prior evidence of its involvement in cell fate regulation during IRI, as well as predicted binding interactions with miR-144-3p. Moreover, miRNAs are increasingly recognized as pivotal regulators of renal ischemia-reperfusion injury. For instance, miR-181d-5p was shown to mitigate tubular apoptosis and inflammatory responses in a mouse IRI model by targeting KLF6, demonstrating the broader relevance of miRNA-mediated modulation in protecting renal function. These studies collectively underscore the significance of miRNA–*EZH2* regulatory networks in controlling renal epithelial survival and the pathogenesis of AKI (41). While this study focused on the *EZH2*-mediated apoptotic pathway, we acknowledge that miR-144-3p may also regulate other injury-relevant mechanisms, such as inflammation and oxidative stress. Emerging evidence further supports the involvement of *EZH2* in acute kidney injury. Li et al. reported that *EZH2* expression is upregulated in sepsis-induced AKI, and its silencing alleviates tubular epithelial apoptosis and inflammation, thereby preserving renal function. This aligns with our findings, highlighting a conserved role for *EZH2* in mediating renal epithelial cell injury across different AKI models (35). Further studies are required to comprehensively explore these alternative downstream pathways and fully elucidate the multifaceted role of miR-144-3p in renal IRI.

Several studies have highlighted the critical roles of the reciprocal regulation between miR-144-3p and *EZH2* in governing cell proliferation, migration, tumorigenesis, invasion, and metastasis in various cancers, including human osteosarcoma, breast cancer, and oral squamous cell carcinoma (42, 43). Generally, miRNAs post-transcriptionally regulate gene expression in animals by partially base-pairing with the 3′ untranslated region (UTR) of target mRNAs, thereby influencing protein synthesis or mRNA stability (44). *EZH2* has been identified as a potential target of miR-144 within the competing endogenous RNA network, with studies validating that it directly binds to the 3′-UTR of *EZH2* to regulate its expression in colorectal cancer (45). Herein, *EZH2* expression was significantly upregulated in both mouse renal ischemia-reperfusion (I/R) tissues and NRK-52E cells under hypoxia/reoxygenation conditions. Overexpression of miR-144-3p suppressed *EZH2* expression in NRK-52E cells under similar conditions. Furthermore, luciferase reporter assays demonstrated that miR-144-3p regulates *EZH2* expression by directly targeting its 3′-UTR. To the best of our knowledge, this is the first study to identify the regulatory relationship between miR-144-3p and *EZH2* in renal I/R injury, providing novel insights into the molecular mechanism by which miR-144-3p modulates *EZH2* expression.

With respect to the translational relevance of our findings, it is noteworthy that the mature sequence of miR-144-3p is identical across human (hsa-miR-144-3p, MIMAT0000436), mouse (mmu-miR-144-3p, MIMAT0000156), and rat (rno-miR-144-3p, MIMAT0000850) (miRBase v22.1) (Supplementary Figure 1D). Furthermore, TargetScan (v8.0) predicts the presence of conserved miR-144-3p binding sites in the *EZH2* 3′UTR across these three species, suggesting that the miR-144-3p/*EZH2* regulatory interaction is evolutionarily conserved (Supplementary Figure 1E). Importantly, we experimentally validated this conservation in human renal proximal tubular epithelial HK-2 cells, where miR-144-3p was significantly downregulated following H/R treatment and miR-144-3p overexpression attenuated apoptosis while enhancing cell viability, phenocopying the protective effects observed in rat NRK-52E cells. Consistently, miR-144-3p has been experimentally validated to target *EZH2* in human cancer cell lines (42, 43, 45, 46), and our current findings in rat NRK-52E cells and mouse kidney tissues further support this conserved targeting relationship. However, it should be noted that the *EZH2* 3′UTR length and the precise number and context of miRNA binding sites may vary across species, and the downstream transcriptional programs regulated by *EZH2* could differ. Therefore, while our findings in rodent models provide a foundation for understanding the miR-144-3p/*EZH2* axis in renal IRI, the direct extrapolation to human pathophysiology should be made with caution pending validation in human-derived experimental systems.

### 4.1 Limitations

Several limitations of the present study should be acknowledged. First, while our dual-luciferase reporter assay demonstrates that *EZH2* knockdown reduces *Bax* and *Caspase-3* promoter activity, the precise molecular mechanism underlying this regulation—whether it involves canonical PRC2/H3K27me3-mediated repression of upstream repressors, non-canonical co-activator functions of *EZH2*, or other indirect pathways—remains to be determined. Future ChIP-based analyses are required to clarify the epigenetic basis of this regulation. Second, the *in vivo* experiments were conducted in C57BL/6 mice, while the *in vitro* studies employed the rat-derived NRK-52E cell line. Although the mature sequence of miR-144-3p is identical across human, mouse, and rat (5′-UACAGUAUAGAUGAUGUACU-3′; miRBase accession: MIMAT0000436, MIMAT0000156, and MIMAT0000850, respectively), and the miR-144-3p binding site within the *EZH2* 3′UTR is conserved among these species as predicted by TargetScan, potential species-specific differences in the downstream regulatory networks involving *EZH2* cannot be excluded. The use of cross-species models limits the direct translational extrapolation of our findings to human renal IRI. Although we have validated key findings in human renal tubular epithelial cells (HK-2)—including miR-144-3p downregulation under H/R, anti-apoptotic effects of miR-144-3p, and *EZH2*-dependent reversal—validation in human renal tissue specimens is still warranted to confirm clinical translatability. Third, this study primarily focused on apoptosis and proliferation as the main phenotypic readouts. Although we assessed oxidative stress markers (MDA and SOD) in the I/R model, we did not comprehensively examine inflammatory responses, mitochondrial dysfunction, or tubular injury markers (e.g., KIM-1, NGAL) in the context of miR-144-3p/*EZH2* modulation. Given the established role of *EZH2* in regulating inflammation in AKI (35), the contribution of the miR-144-3p/*EZH2* axis to these additional injury phenotypes merits dedicated investigation. Fourth, the *in vivo* miR-144-3p mimics were administered prior to I/R injury, which provides proof-of-concept for a preventive strategy but does not address the therapeutic potential of post-injury intervention, which is more clinically relevant for AKI management. Fifth, the sample size for *in vivo* experiments (*n* = 6 per group), while adequate for detecting moderate-to-large effect sizes, may be underpowered for detecting subtle phenotypic differences, and independent replication in larger cohorts would strengthen the conclusions.

## 5 Conclusion

In summary, this study identified miR-144-3p as a protective regulator in renal ischemia-reperfusion injury. It was significantly downregulated under I/R conditions and mitigated cellular injury by suppressing *EZH2* expression through direct binding to its 3′UTR, thereby reducing the levels of pro-apoptotic proteins Bax and Caspase-3. Notably, *EZH2* knockdown reduced the promoter activity and protein expression of Bax and Caspase-3, indicating that *EZH2* positively regulates the transcription of these pro-apoptotic genes, likely through an indirect or non-canonical mechanism. These findings expand our understanding of miRNA-mediated regulatory mechanisms in renal I/R and highlight the miR-144-3p/*EZH2* axis as a promising therapeutic target. Future studies are warranted to elucidate the precise molecular mechanism by which *EZH2* regulates *Bax* and *Caspase-3* transcription and to validate these findings in human-derived experimental systems.

## Data Availability

The raw data supporting the conclusions of this article will be made available by the authors, without undue reservation.
