# Supplementary figures and images for: MiR-144-3p regulates cell proliferation and apoptosis in renal ischemia-reperfusion injury by targeting EZH2
Source: Front Med (Lausanne). 2026 Jul 2;13:1745179. doi: 10.3389/fmed.2026.1745179 (PMC13383657; doi:10.3389/fmed.2026.1745179)

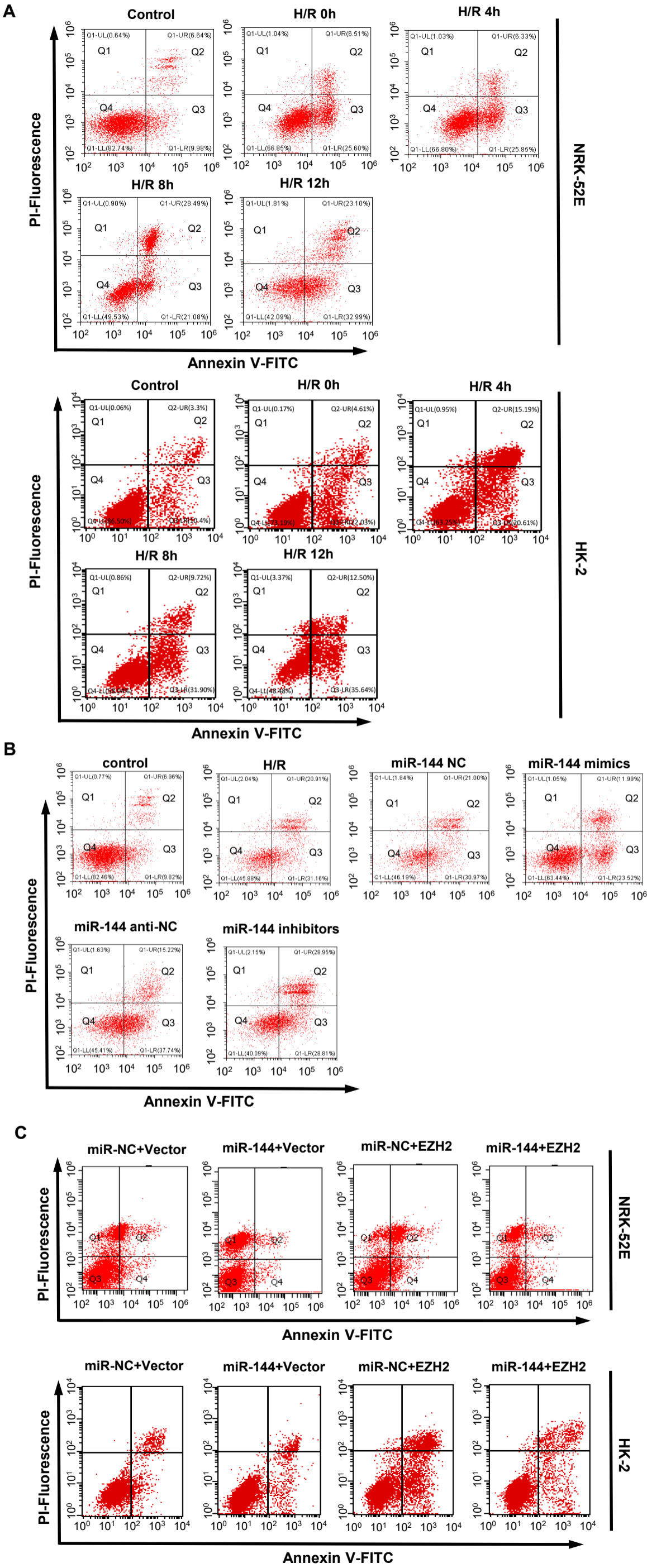

Supplement: Supplementary file 1 [file Data_Sheet_1.pdf]
